# Independent and joint effects of moderate alcohol consumption and smoking on the risks of non-alcoholic fatty liver disease in elderly Chinese men

**DOI:** 10.1371/journal.pone.0181497

**Published:** 2017-07-20

**Authors:** Peiyi Liu, Yanyan Xu, Yuhan Tang, Min Du, Xiao Yu, Jian Sun, Lin Xiao, Meian He, Sheng Wei, Jing Yuan, Youjie Wang, Yuan Liang, Tangchun Wu, Xiaoping Miao, Ping Yao

**Affiliations:** 1 Department of Nutrition and Food Hygiene, Hubei Key Laboratory of Food Nutrition and Safety and the Ministry of Education (MOE) Key Lab of Environment and Health, School of Public Health, Tongji Medical College, Huazhong University of Science and Technology, Wuhan, Hubei, China; 2 Institute of Occupational Medicine and the Ministry of Education (MOE) Key Lab of Environment and Health, School of Public Health, Tongji Medical College, Huazhong University of Science and Technology, Wuhan, Hubei, China; 3 Department of Epidemiology and Biostatistics and the Ministry of Education (MOE) Key Lab of Environment and Health, School of Public Health, Tongji Medical College, Huazhong University of Science and Technology, Wuhan, Hubei, China; Medizinische Fakultat der RWTH Aachen, GERMANY

## Abstract

**Background:**

Whether cigarette smoking and moderate drinking are associated with non-alcoholic fatty liver disease (NAFLD)has not been fully described. This study investigated the separate and joint effects of smoking and moderate drinking on Chinese men with NAFLD.

**Methods:**

Across-sectional assay from DFTJ Cohort study was performed with a size of 9432 elderly Chinese men excluding excessive alcohol consumption (<210g/week). Fatty liver was diagnosed by standardized ultrasonographic inspection. The odds ratio (OR) of alcohol consumption and smoking for the prevalence of NAFLD were analyzed using multiple logistic regression with multiple adjustments.

**Results:**

The prevalence of NAFLD in current smokers (pack-year≥40) and drinkers (80~210g/week or drinking duration≥35years) was significantly higher than that in non-smokers and non-drinkers, respectively. The combination of current smoking (pack-year≥40) and drinking (80~210g/week) was associated with the highest risk of NAFLD (OR 1.85; 95% confidence interval [CI] 1.28–2.68;*P*<0.01). The similar combined effect was found in participants with pack-year≥40 and drinking duration≥35 years (OR 1.72; 95% CI 1.26–2.34;*P*<0.01). Moreover, an interaction was observed between current smoking and moderate drinking in NAFLD.

**Conclusions:**

In elderly Chinese men, cigarette smoking and moderate alcohol consumption exerts an evident joint effect and interaction on the prevalence of NAFLD, although both are significantly and independently associated with NAFLD prevalence. Such findings highlight particular significance of avoidance of cigarette and alcohol on NAFLD prevention.

## Introduction

Nonalcoholic fatty liver disease (NAFLD) is becoming the leading cause of chronic liver disease in the Western world with an estimated prevalence of 20%~30%[1].Excessive alcohol consumption has been absolutely regarded as a major etiology of hepatic steatosis and liver injury[2]; nevertheless, mild or moderate alcohol consumption, has attracted increasing attention for its equivocal healthy consequences. For instance, a number of cross-sectional studies reported that moderate or light alcohol consumption played a protective role against fatty liver [3–7]. Similar findings were demonstrated by several longitudinal research [8,9]. Also some clinical reports showed that light to moderate alcohol consumption was inversely associated with severity of NAFLD[4,10]. On the other hand, increased gamma-glutamyl transpeptidase (GGT) in patients with fatty liver was observed even though alcohol consumption was lower than 60g per week[11].Wang et al [12]found that modest alcohol consumption increased hepatic fat accumulation among Hong Kong Chinese, in spite of unchanged risk of advanced fibrosis. In addition, among patients with NASH cirrhosis followed prospectively, moderate alcohol use was associated with an increased risk of hepatocarcinoma (HCC), (Hazard ratio [HR]: 3.8, 95%confidence interval[CI]: 1.6–8.9,*P* = 0.002)[13].Given the accumulating contradictory epidemiological studies on the healthy consequences of alcohol consumption at mild or moderate level, and the neglect on long-term healthy outcomes of moderate drinking by most studies, the suggestion on alcohol consumption is still equivocal to some extent.

Alcohol consumption often co-occurs with cigarette smoking. Similar with alcohol abuse, cigarette smoking has been regarded as another widely accepted and avoidable behavioral risk factors for premature morbidity and death. Of note, cigarette smokers frequently weigh less than persons of the same sex and age who never smoked [14], implying smoking could reduce the risk of NAFLD. However, most of epidemiological investigations showed smoking could be served as a risk factor of NAFLD[15]. Besides NAFLD, insulin resistance, diabetes, dyslipidemia, and etc, has been reported to link with cigarette smoking [16,17]. It is not clear, to date, whether there is a synergistic effect between smoking and moderate drinking on NAFLD, and how they collectively affect NAFLD onset.

Thus, the present cross-sectional assay from DFTJ Cohort study was performed to identify the independent and combined effects of smoking intensity and the duration of light-to-moderate drinking on the prevalence of NAFLD in male population in China.

## Materials and methods

### Study population

The data was collected from DFTJ Cohort study which was launched in 2008 among retirees of Dongf eng Motor Corporation (DMC) in Shiyan City, Hubei province[18]. DMC was founded in 1969 and became one of the three largest auto manufacturers in China.

**Fig 1:**
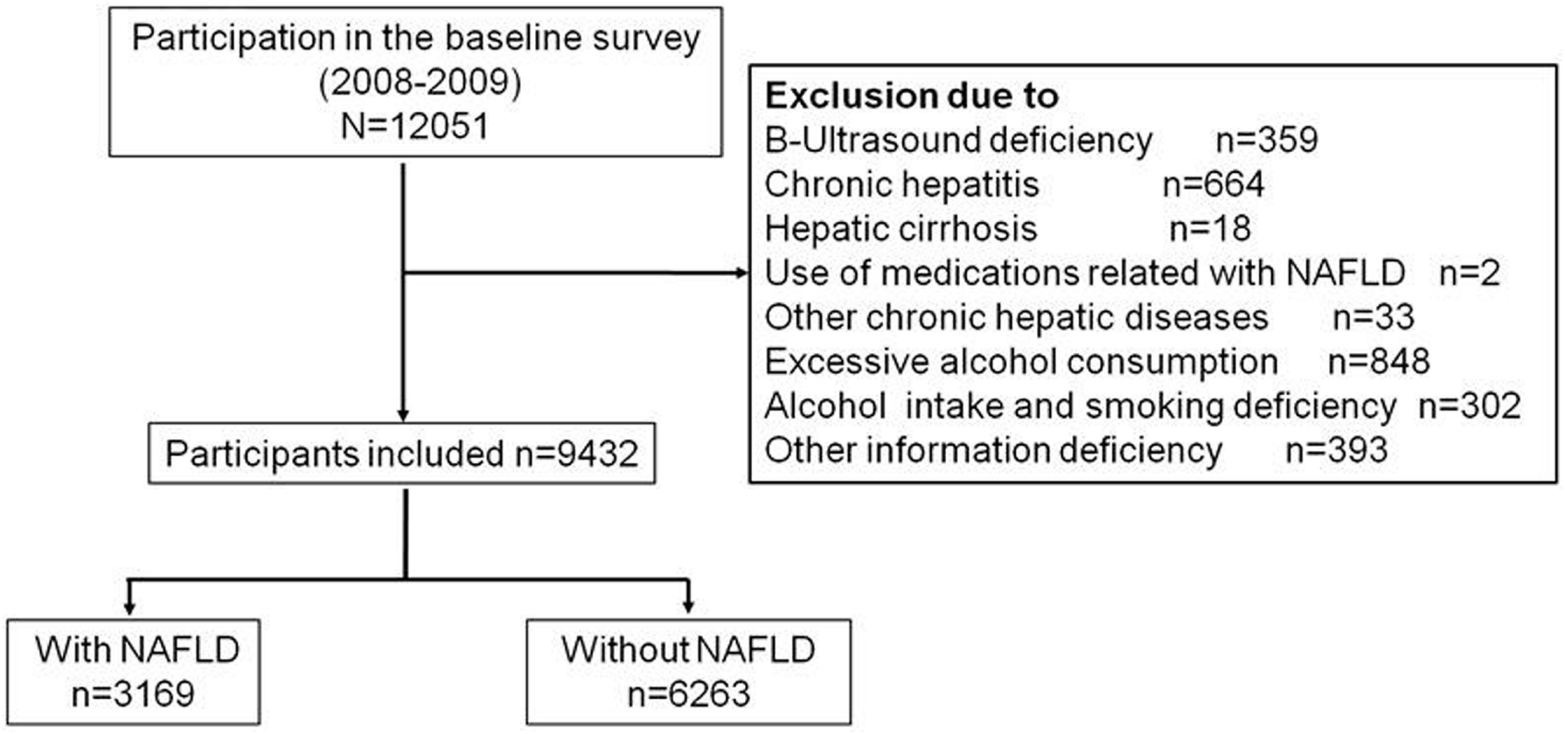
Flowchart of our cross-sectional study.

This study focused on a sample consisting of male retirees, who reported undergoing health checkup and questionnaires (n = 12051). Subjects enrolled in the study underwent ultrasonography as a part ofthe systemic health checkup during 2008 to 2010(n = 11692). Subjects at baseline with presence of any of following conditions were excluded: chronic hepatitis (n = 664), hepatic cirrhosis (n = 18), usage of medications (such as valproate, amiodarone or tamoxifen) with known hepatotoxicity within the past two weeks(n = 2),other chronic hepatic diseases (including carcinoma and schistosome hepatic disease, n = 33), excessive alcohol consumption (more than 210g per week, n = 848), information incompletion of age, BMI, smoking or drinking (n = 695).Finally, the eligible sample size for analyses was 9432 in the present study (Fig 1).

### Data collection

Baseline data was collected by trained interviewers via semi-structured questionnaires during face-to-face interviews. The questionnaire was designed based on 6 pilot surveys among this population. Information on socio-demographic factors, health status, and lifestyle practices (including smoking and physical activity) was included in the questionnaires.

All subjects were screened for NAFLD by a single, expert radiologist using a sensitive ultrasound machine (Aplio XG, TOSHIBA, Japan). Fatty liver was diagnosed in the presence of diffuse increase of hepatic echogenicity and decrease in density, accompanying unclear display of intra-hepatic lacuna structure or mild to moderate hepatomegaly with a round and blunt border.

Drinking status was classified into three groups: non-drinking, current drinking (including moderate drinkers and excessive drinkers) and ex-drinking. Those who drank more than once a week last half a year were current drinkers. Non-drinkers were defined as those who consumed less than this predetermined frequency. Ex-drinkers were defined as people who used to drink but didn’t drink no more in the last 6 months. Moderate drinking was defined as alcohol consumption up to an average of 210 gram per week. The information obtained in the questionnaire included frequency of drinking per week and average amounts of alcohol consumed at a time. The amount of alcohol consumed (mL) every time was reported separately as beer, wine and hard liquor, and weekly consumption was calculated according to their average alcoholicity (beer 4%, wine 12%, and hard liquor 42%, respectively). Drinking duration (period of drinking) equals age minus beginning-to-drinking age. The categorization was based on quartile of alcohol consumption and median duration of drinking in our population. Never drinking (non-drinkers), not including ex-drinkers, were considered as the reference group in multiple logistical regression model analysis.

According to the smoking status from the respondents’ self-report, participants were grouped as current smokers, ex-smokers, and non-smokers. Current smoking was defined as that who had smoked at least one cigarette per day for more than half of year. Ex-smoker was defined as who had a cessation of smoking >6 months. Current smoking was categorized by pack-year(number of packs smoked per day multiplied by the number of years smoked).Other variables were dichotomized as yes or no on the basis of the responses to questions on the current use of the antihypertensive drug, aspirin, and antibiotics, and past history of coronary heart diseases (CHD), diabetes mellitus, hypertension and stroke. Frequency and the average duration of each type of physical activity were obtained via the questionnaires. According to the participants’ self-reported responses, physical activity was dichotomized as yes or no.

All subjects were examined in the morning after an overnight fast. Blood pressure was measured by a mercury sphygmomanometer in the morning. Fasting blood was drawn with a vacuum coagulation tube for serum, with five milliliters in the tube. Serum triglyceride (TG), uric acid (UA), total cholesterol (TC), blood glucose, alanine aminotransferase (ALT), aspartate aminotransferase (AST), low-density lipoprotein (LDL) cholesterol and high-density lipoprotein (HDL) cholesterol were measured by the hospital’s laboratory using ARCHITECTci8200, Abbott, USA.

### Ethical considerations

The study was approved by the Medical Ethics Committee of the School of Public Health, Tongji Medical College, and Dongfeng General Hospital, DMC. All participants provided written informed consent.

### Statistical analysis

All statistical analyses were performed using SAS 9.2 software. Continuous variables were expressed as mean± standard deviation (SD) and were analyzed by One-Way variance or Student’s t-test. Categorical variables were expressed as percentages (numbers) and compared using Pearson’s chi-squared test. Multivariable logistic regression analyses were used to adjust the influence of alcohol consumption and smoking for other confounders determined by univariate analysis. All variables, such as age, BMI, waist, diabetes status, and etc. that were potentially implicated in NAFLD, drink or smoking, were included. Other variables with statistical significance in the unadjusted analyses were also introduced. The multivariate adjustment in the model b included age (continuous), BMI(continuous), waist(continuous), AST/ALT(continuous), TG (continuous), TC (continuous), HDL (continuous), LDL (continuous), UA (continuous), systolic blood pressure (SBP, continuous), diastolic blood pressure (DBP, continuous), physical activity (yes/no), diabetes, past history (yes/no) of CHD and hypertension, and cigarette smoking or alcohol consumption status. The interaction between alcohol and smoking were calculated by setting dummy variables with nondrinker and nonsmoker as references, according to the method described by Andersson [19].All *P* values were two-tailed, and statistical significance was set at *P*< 0.05.

## Results

### Subject description at baseline

Baseline characteristics of the population enrolled are shown in Table 1 according to the status of NAFLD. Totally, the overall prevalence of NAFLD was 33.60%among 9432male participants in 2008 to 2010. The prevalence of NAFLD in current drinkers, ex-drinkers, and non-drinkers were 35.10%, 35.13% and 32.35%, respectively (S1 Table). The prevalence of NAFLD in current smokers, ex-smokers, and non-smokers were 33.24%, 36.16% and 32.29%, respectively (S2 Table). Participants with NAFLD showed a lack of physical activity, had a higher BMI and waist circumference compared to men without NAFLD (*P*< 0.0001).The mean age of cases with NAFLD was significantly lower than those without NAFLD (*P*<0.0001). As expected, the mean of ASL/ALT levels was also significantly lower in NAFLD (+) cases than in NAFLD (−) cases (1.01 vs. 1.24, *P*<0.0001). The assay on physiological and metabolic parameters displayed that NAFLD (+) cases had significantly higher level on uric acid, systolic blood pressure, diastolic blood pressure, LDL, TG, TC and a lower HDL was significantly lower in NAFLD (+) cases compared with that in NAFLD (−) cases (all *P*<0.0001). Accordingly, the presence of metabolic abnormalities, including hypertension, CHD or diabetes mellitus, was more frequent in NAFLD (+) cases than that in NAFLD (−) subjects (all *P*<0.0001). Notably, the distribution of alcohol consumption and smoking categories was significantly different between NAFLD (+) and NAFLD (−) cases (all *P*<0.0001).

**Table 1:** Baseline characteristics of study participants (n = 9432).

	NAFLD (+)	NAFLD (−)	*P* value
Total number	3169	6263	
Age(years)	65.02(6.80)	65.89(6.78)	<0.0001
Body Mass Index(kg/m^2^)	26.68(2.82)	23.56(2.87)	<0.0001
Overweight, n(%)	2679(84.54)	2791(44.56)	<0.0001
Education(1/2/3)	26.43/35.86/37.71	28.95/33.89/37.16	0.0270
Waist circumference(cm)	90.68(8.40)	82.51(9.10)	<0.0001
HDL (mmol/l)	1.28(0.38)	1.38(0.40)	<0.0001
LDL (mmol/l)	3.08(1.01)	2.94(0.79)	<0.0001
ALT (U/L)	29.98(22.36)	22.86(22.59)	<0.0001
AST (U/L)	27.10(20.70)	24.92(13.55)	<0.0001
AST/ALT	1.01(0.41)	1.24(0.52)	<0.0001
Triglyceride (mmol/l)	1.89(1.72)	1.19(0.78)	<0.0001
Cholesterol (mmol/l)	5.14(1.02)	4.89(0.92)	<0.0001
Uric Acid (μmol/l)	351.30(84.58)	317.34(77.85)	<0.0001
Systolic blood pressure (mm Hg)	133.32(17.58)	130.39(18.43)	<0.0001
Diastolic blood pressure (mm Hg)	79.77(10.90)	77.43(11.01)	<0.0001
Diabetes Mellitus, n(%)	955(30.15)	986(15.75)	<0.0001
History of coronary heart disease, n(%)	1014(32.02)	1488(23.77)	<0.0001
History of hypertension, n(%)	1679(53.02)	2342(37.40)	<0.0001
History of stroke, n(%)	211(6.66)	365(5.83)	0.1091
Physical activity, n(%)	2645(83.46)	5352(85.45)	0.0111
Drink status			
Non-drinker	1675(52.86)	3502(55.29)	
Ex-drinker	417(13.16)	770(12.29)	
Current drinker	1077(33.79)	1991(31.79)	0.0187
Smoke status			
Non-smoker	1235(38.97)	2590(41.35)	
Ex-smoker	869(27.42)	1534(24.49)	
Current smoker	1065(33.61)	2139(34.15)	0.0060

One-Way variance test for continuous variables or Pearson’s qui-squared test for categorical variables. Overweight was defined as BMI≥24. Educational was categorized as1, 2 and 3, which mean low (0 to 6 years), medium (7 to 9 years), and high (≥10 years) respectively.

### Separate and joint effects of moderate alcohol consumption and cigarette smoking on the prevalence of NAFLD

Table 2 shows a independent association between NAFLD and alcohol intake or cigarette smoking. The multivariate-adjusted odds ratios (ORs) for NAFLD prevalence in those who consumed 80 ~159.9 grams and 160 ~ 210 grams ethanol per week were 1.20 (95% CI: 1.02 to 1.41) and 1.43(95% CI:1.15 to1.78) respectively, after adjusting age, BMI, waist and diabetes. Such association kept unchanged after further adjustment in model b, including smoking, AST/ALT, HDL, LDL, DBP, SBP, TG, TC, UA, physical activity, past history of CHD and hypertension (OR:1.30, 95% CI:1.07 to 1.59; OR: 1.46, 95% CI: 1.12 to 1.89). Significantly, the odd of NAFLD was 1.32 times (95% CI: 1.13 to 1.53) higher in current drinkers with drinking duration≥35 years than non-drinkers in the model b, indicating a robust association between alcohol consumption and NAFLD risk.

**Table 2:** Multiple logistic regression analysis for NAFLD in the cross-sectional study (n = 9432).

	n	Prevalence of NAFLD(%)	Unadjusted OR and 95% CI	Multivariated OR^a^ and 95% CI	Multivariate OR^b^ and 95% CI
Alcohol intake					
Non-drinker	5177	32.35	reference	reference	reference
Current drinker	3068				
0.1~79.9g/week	1482	34.55	1.10(0.98-1.25)	1.15(1.00-1.33)	1.15(0.97-1.36)
80~159.9g/week	1060	34.81	1/12(0.97-1.28)	1.20(1.02-1.41)^*^	1.30(1.07-1.59)^**^
160~210g/week	526	37.26	1.24(1.03-1.50)^*^	1.43(1.15-1.78)^**^	1.46(1.12-1.89)^**^
1~34 year	981	32.11	0.99(0.85-1.14)	1.06(0.89-1.26)	1.09(0.89-1.34)^*^
≥35year	2087	36.51	1.20(1.08-1.34)^**^	1.28(1.13-1.45)^**^	1.32(1.13-1.53)^**^
Ex-drinker	1187	35.13	1.13(0.99-1.29)	0.96(0.82-1.21)	0.96(0.80-1.17)
Smoking					
Non-smoker	3825	32.29	reference	reference	reference
Current smoker	3204				
Pack-year 1~19.9	1029	32.17	1.00(0.86-1.15)	1.15(0.97-1.37)	1.19(0.97-1.47)
Pack-year 20~39.9	1244	32.07	0.99(0.86-1.14)	1.23(1.05-1.45)^*^	1.22(1.00-1.49)^*^
Pack-year ≥40	931	35.98	1.18(1.02-1.37)	1.41(1.18-1.68)^**^	1.52(1.22-1.88)^**^
Ex-smoker	2403	36.16	1.19(1.07-1.32)^**^	1.16(1.02-1.31)^*^	1.12(0.96-1.31)

^a^ Adjusted for the age, BMI, waist and diabetes.

^b^ Adjusted for model a plus AST/ALT, HDL, LDL, DBP, SBP, TG, TC, UA, physical activity, past history of CHD and hypertension, smoking (non-smoking, current smoking, ex-smoking) or drink (non-drinking, current drinking and ex-drinking).

* *P*<0.05

** *P*<0.01

Interaction = 1.60(1.33–1.93), *P*<0.0001, the reference was non-drinker and non-smoker.

As for the influence of smoking on NAFLD, a multivariate logistic regression model showed the adjusted ORs of subjects with pack-year between 20 and 40 and more than 40 were 1.23 (95% CI: 1.05 to1.45) and 1.41 (95% CI: 1.18 to1.68)compared to non-smokers respectively, when adjusted for age, BMI, waist and diabetes (Table 2).Furthermore, both current smokers with pack-year between 20 and 40 and those with pack-year more than 40 came with higher risks of NAFLD than that in non-smokers. Meanwhile, the ratios were not further attenuated after additional adjustment in model b. The ex-smokers also had an increased prevalence of NAFLD than non-smokers by crude analysis with first model. However, the fully adjusted OR of NAFLD prevalence was 1.12(95% CI:0.96 to 1.31, *P*> 0.05) in ex-smokers compared with non-smokers. Moreover, an evident interaction between smoking and drink came to prominence to synergistically promote NAFLD (Interaction = 1.60(1.33 to 1.93), *P*<0.0001).

To further explore the synergistic effect, stratified analysis was performed to evaluate the combined effects of moderate alcohol drinking and cigarette smoking on the risk of NAFLD following the exclusion of ex-smokers and ex-drinkers. As shown in Table 3 focusing on the joint effect of smoking and drinking amount, the ORs of the prevalence of NAFLD in subjects who smoked ≥40 pack-year were 1.60 (95% CI:1.16 to 2.20) for non-drinker, 1.73 (1.15 to 2.60) for light drinker < 80 g/week, and 1.85 (1.28 to 2.68) for moderate drinker (80 ~ 210 g/week) with participants who neither smoked nor drank alcohol as reference, after adjusting age, BMI, waist, diabetes, AST/ALT, HDL, LDL, DBP, SBP, TG, TC, UA, physical activity, past history of CHD and hypertension. Even though the smoking was less than 40 pack-year, light and moderate drinking still increased the risk of NAFLD after adjusting multiple variables (OR: 1.38, 95% CI: 1.05 to 1.80 for light drinking; OR: 1.68, 95% CI: 1.28 to 2.20 for moderate drinking) when compared with non-drinkers and non-smokers.

**Table 3:** Adjusted odds ratios of NAFLD according to combined cigarette smoking and weekly alcohol consumption(n = 6496).

		Non-drinker	Alcohol consumption	
			0.1~79.9g/week	80~210g/week
Non-smoker	N	2918	379	340
	NAFLD	925(31.70)	123(32.45)	119(35.00)
	OR(95% CI)	1.00 reference	1.12(0.83-1.52)	1.18(0.86-1.64)
Pack-year				
1~39.9	N	925	532	584
	NAFLD	282(30.34)	178(33.46)	196(33.56)
	OR(95% CI)	1.12(0.90-1.40)	1.38(1.05-1.80)^*^	1.68(1.28-2.20)^**^
≥40	N	372	180	266
	NAFLD	127(34.14)	67(37.22)	95(35.71)
	OR(95% CI)	1.60(1.16-2.20)^**^	1.73(1.15-2.60)^**^	1.85(1.28-2.68)^**^

The multiple logistic regression analysis were used for prevalence of NAFLD adjusted for age, BMI, waist, diabetes, AST/ALT,HDL, LDL, DBP, SBP, TG, TC, UA,physical activity, past historyof CHDand hypertension. The test excluded ex-smokers and ex-drinkers.

^*^P<0.05

^**^P<0.01

Subsequently, the joint effects between drinking duration and cigarette smoking on the prevalence of NAFLD are shown in Table 4. Compared with non-drinkers and non-smokers, current smokers with more than 40 pack-year had a significantly higher risk of NAFLD, regardless of whether they drank or not. Current drinkers who smoked less than 40 pack-year with drinking duration <35 years and ≥ 35 years had ORs of1.45 (95%CI:1.06–2.00) and 1.55 (95% CI:1.22–1.97) in comparison with those neither drank nor smoked, respectively. Of note, the prevalence of NAFLD in current smokers with more than 40 pack-year was gradually increased with drinking duration (34.14%, 34.44% and 36.80%);however, the risk of NAFLD failed to linearly increase with drinking duration when adjusted for age, BMI, waist, diabetes, AST/ALT, HDL, LDL, DBP, SBP, TG, TC, UA, physical activity, past history of CHD and hypertension.

**Table 4:** Adjusted odds ratios of NAFLD according to combined cigarette smoking and alcohol consumption duration (n = 6496).

		Non-drinker	Duration of drinking	
			0.1~34 year	≥35 year
Non-smoker	N	2918	293	426
	NAFLD	925(31.70)	86(29.35)	156(36.62)
	OR(95% CI)	1.00 reference	0.89(0.62-1.28)	1.34(1.01-1.79)^*^
Pack-year				
1~39.9	N	925	403	713
	NAFLD	282(30.49)	134(33.25)	240(33.66)
	OR(95% CI)	1.11(0.89-1.39)	1.45(1.06-2.00)^*^	1.55(1.22-1.97)^**^
≥40	N	372	90	356
	NAFLD	127(34.14)	31(34.44)	131(36.80)
	OR(95% CI)	1.60(1.16-2.20)^**^	2.14(1.17-3.91)^*^	1.72(1.26-2.34)^**^

The multiple logistic regression analysis were used for prevalence of NAFLD adjusted for age, BMI, waist, diabetes, AST/ALT, HDL, LDL, DBP, SBP, TG, TC, UA,physical activity, past history of CHDand hypertension. The test excluded ex-smokers and ex-drinkers.

^*^P<0.05

^**^P<0.01

## Discussion

This study showed a clear evidence that both moderate alcohol consumption and cigarette smoking were positively associated with the risk of NAFLD at a manner of exposure-response relationship. Moreover, we found a mutual enhancement by moderate alcohol consumption and smoking on the risk of NAFLD in elderly Chinese men. In particular, the combination of smoking and chronic moderate drinking was strongly associated with the prevalence of NAFLD.

Alcohol has long been thought to cause fatty liver through altering hepatic NADH/NAD^+^ redox potential, which, in turn, inhibits fatty acid oxidation and the activity of tricarboxylic acid cycle reactions. Emerging studies indicate that additional effects of ethanol both impair fat oxidation and stimulate lipogenesis [20]. However, a mass of studies showed that the proportion of NAFLD was lower in light or moderate alcohol drinkers than non-drinkers [3–9]. The plausible mechanistic explanation may be related to activating AMPK pathway and/or PPAR-αpathway and/or repressingSREBP-1c pathway[9]. Recently, the views about protective effects of moderate alcohol consumption on healthy consequence have also been questioned. Several cohort studies have demonstrated that moderate alcohol use is not beneficial for heart function via QT interval or heart rate in men[21], but associated with higher fasting glucose[22]as well as increased alcohol related cancers in women[23]. In our study, long-term drinking (≥ 35 years)even with alleged moderate rang (80–210 g/week), was still a risk factor for NAFLD in elder men (Table 2). The discrepancy may be attributed: First, the genetic difference of alcohol metabolism among different populations may contribute to the different healthy consequence. Genetic variations of genes encoding the two enzymes alcohol dehydrogenase (ADH) and acetaldehyde dehydrogenase (ALDH) are very common in East Asians but relatively rare in most other populations, indicating a low ethanol metabolism capacity in our subjects. Secondly, the average ages of population in previous studies are from 39.2 to 50.9 years. In our study, the average age of subjects was 65.6 years old and average drinking duration was 37.1 years, which was conducive to the check of long-term effects of chronic “safe” consumption. Thirdly, the activity of enzyme ADH, as well as the loss of lean body mass, is significantly reduced in older adults[24], potentially increasing the amount of ethanol in blood-stream[25].Therefore, older adults may be more vulnerable to alcohol consumption, even at relatively low levels. Thus, the association between long-term effect of moderate alcohol consumption and the prevalence of NAFLD remind us to be more cautious about once-alleged “healthy” consumption of alcohol.

Previous studies have confirmed that cigarette smoking is associated with chronic liver diseases such as chronic hepatitis C and B, alcoholic liver diseases[26–28], and NAFLD [29,30]. Cigarette smoking is a risk factor in NAFLD, and amplified the risk of NAFLD by interacting with gene genotypes of adiponectin gene and glutathione peroxidase-1 (GPx-1) gene[29].Our data also showed the prevalence of NAFLD was increased with pack-year. Long-term smoking can cause oxidation of glucose metabolism in cells, and increase free fatty acids levels in plasma, and which can be taken up by liver and adipose tissue to synthesize triglycerides, leading to the development of insulin resistance and NAFLD. In addition, the nicotine in tobacco can cause sympathetic excitement and increase the release of catecholamines and glucagon, exerting the first hit in the pathogenesis of NAFLD[31,32].

Given that alcohol consumption and smoking are frequently coexist in Chinese men but their combined or interactive effects on NAFLD have not fully be elucidated, a further analysis was performed and our data showed a multiplicative interaction of smoking and moderate drinking in association with NAFLD. The similar model was found in metabolic syndrome in Japanese and Chinese men [30,33], as well as cirrhosis in middle-aged women [34], although alcohol consumption exceeded the range of moderate drinking. As compared with nonsmokers, male smokers tend to exercise less regularly, drink more alcohol, consume more salty foods, and eat more quickly. Furthermore, male daily drinkers are likely to intake a greater number of salty foods, more fat, and less fruit than others [30,35].The reason for the marked increase in the OR for this combination, which was particularly prominent in heavy smokers, appears to be the synergism of accumulated unhealthy behaviors. The study from Chinese men has observed there is an interaction of current smoking with former alcohol consumption on the increased prevalence of metabolic syndrome and central obesity [33]. And the interaction of smoking ≥30 cigarettes per day with drinking ≥69 grams ethanol per day on the increased prevalence of metabolic syndrome in Japanese men has also been observed [30]. Therefore, our findings suggest that, both smoking and chronic alcohol consumption, even at moderate level, are the risk but not beneficial factor of NAFLD. NAFLD prevalence may be higher as a result of coexistence of smoking and drinking because of their joint effect with multiplicative interaction.

The strength of our study is that we comprehensively analyzed the separate and particularly joint association between two risk factors (smoking and drinking, specially at moderate level),with the prevalence of NAFLD. Furthermore, we found that concomitant smoking and moderate drinking are associated with a higher prevalence of NAFLD. In addition, we took multiple confounding factors into account in the analysis, such as age, BMI, waist, the ratio of AST and ALT, diabetes, concentrate of HDL, LDL, UA, TG and TC, levels of DBP, SBP, physical activity and past history of related diseases. This may be more plausible for causal relationship between cigarette smoking and/or moderate alcohol intake, and NAFLD.

The limitations of this study included the cross-sectional design and diagnostic method. Larger samples are needed to be included for analysis. In addition, In addition, NAFLD was diagnosed by ultrasonographic method. Actually, liver biopsy is difficult and impractical for NAFLD in community-based research studies[36], despite its role as gold standard for the assessment of liver diseases. Ultrasonographic definition of steatosis has been frequently used in Chinese epidemiological research studies [37,38], and has been endorsed by Asia-Pacific regional guidelines[39]. In spite of imperfect, any misclassification bias based on the ultrasonographic diagnosis should affect drinkers and nondrinkers equally, therefore the bias would only falsely diminish the size of the observed association. Additionally, data on the presence of HBsAg infection was derived from self-reported disease and drug history. Hence, the sample size of participants may be overestimated since data to distinguish participants on the presence or not of HBsAg antibodies at baseline was unavailable. As for precision, we have trained interviewers to deal with data using standardized methodology, although it couldn't fully eliminate errors in the self-reported amount of alcohol and cigarette consumed. In addition, multiple confounding factors, including social, demographic, metabolic parameter and lifestyle covariates, have been entered into the multivariate analysis for reducing the interference of other factors. This minimize, although not entirely rule out, the possibility that self-report of modest drinking is a surrogate marker of another unmeasured lifestyle factor. Even if there was the misclassification of alcohol consumption due to the recall bias or behavior change, the prevalence of non-drinkers and moderate drinkers would be both overestimated. Then the effect of modest drinking on NAFLD remains unchanged and well-documented. Finally, the cross-sectional design has been traditionally difficult to measure the safety of moderate alcohol consumption and smoking in subjects with preexisting NAFLD. So the detrimental potentiality of moderate drinking and smoking should be further explored with prospective cohort and experimental studies.

In conclusion, moderate alcohol consumption and cigarette smoking are independently and synergistically associated with the prevalence of NAFLD in elder Chinese men. Considering the rapid increase in the prevalence of NAFLD and the high proportion of current smokers and drinkers among Chinese adults, the study may highlight the significance of public health for the management of risk factors, although further cohort studies are warranted to validate the roles of cigarette smoking and alcohol intake in the pathogenesis of NAFLD.

## Supporting information

S1 Table Baseline characteristics of study participants according to alcohol consumption (n = 9432). (DOCX)

S2 Table Baseline characteristics of study participants according to cigarette smoking (n = 9432). (DOCX)
