# Supplementary material for: Independent and joint effects of moderate alcohol consumption and smoking on the risks of non-alcoholic fatty liver disease in elderly Chinese men
Source: PLoS One. 2017 Jul 20;12(7):e0181497. doi: 10.1371/journal.pone.0181497 (PMC5519158; doi:10.1371/journal.pone.0181497)
Supplement: S1 Table — (DOCX) [file pone.0181497.s001.docx]

|  | Non-drinker | Moderate drinker | Abstainer | *P* value |
| --- | --- | --- | --- | --- |
| Total number | 5177 | 3068 | 1187 |  |
| Age(years) | 66.33(7.00) | 64.31(6.46) | 65.71(6.28) | <0.0001 |
| Body Mass Index(kg/m^2^) | 24.61(3.25) | 24.47(3.11) | 24.91(3.24) | 0.0002 |
| Overweight, n(%) | 2996(58.87) | 1735(56.55） | 739(62.26) | 0.0016 |
| Education(1/2/3) | 26.02/33.56/40.42 | 29.81/36.13/34.06 | 32.80/34.75/32.46 | <0.0001 |
| Waist circumference(cm) | 85.12(9.63) | 85.00(9.87) | 86.53(9.28) | <0.0001 |
| HDL (mmol/l) | 1.33(0.42) | 1.40(0.38) | 1.30(0.36) | <0.0001 |
| LDL (mmol/l) | 2.97(0.82) | 3.03(0.99) | 2.94(0.81) | 0.0034 |
| ALT (U/L) | 25.57(26.68) | 24.42(14.84) | 26.06(20.90) | 0.0444 |
| AST (U/L) | 25.41(18.11) | 26.14(14.42) | 25.46(12.34) | 0.1486 |
| AST/ALT | 1.15(0.48) | 1.20(0.54) | 1.13(0.47) | <0.0001 |
| Triglyceride (mmol/l) | 1.42(1.08) | 1.44(1.54) | 1.44(0.96) | 0.8269 |
| Cholesterol (mmol/l) | 4.95(0.95) | 5.06(0.98) | 4.89(0.95) | <0.0001 |
| Uric Acid (μmol/l) | 327.26(82.12) | 329.64(79.23) | 333.12(86.35) | 0.0751 |
| Systolic blood pressure (mm Hg) | 131.38(18.27) | 131.43(18.26) | 131.21(17.73) | 0.9394 |
| Diastolic blood pressure (mm Hg) | 70.70(10.85) | 79.17(11.28) | 78.02(10.97) | <0.0001 |
| Diabetes Mellitus, n(%) | 1118(21.60) | 505(16.46) | 318(26.79) | <0.0001 |
| History of coronary heart disease, n(%) | 1390(26.85) | 631(20.59) | 481(40.52) | <0.0001 |
| History of hypertension, n(%) | 2199(42.48) | 1188(38.76) | 634(53.41) | <0.0001 |
| History of stroke, n(%) | 313(6.05) | 124(4.04) | 139(11.71) | <0.0001 |
| NAFLD, n(%) | 1675(32.35) | 1077(35.10) | 417(35.13) | 0.0187 |
| Current smoker, n(%) | 1297(25.05) | 1562(50.91) | 345(29.06) | <0.0001 |
| Physical activity, n(%) | 4365(84.32) | 2591(84.45) | 1041(87.70) | 0.0113 |

Table S1 Baseline characteristics of study participants according to alcohol consumption (n=9432)

One-Way variance test for continuous variables or Pearson’s qui-squared test for categorical variables.

Overweight was defined as BMI≥24.

Educational was categorized as1, 2 and 3, which mean low (0 to 6 years), medium (7 to 9 years), and high (≥10 years) respectively.
