# Supplementary material for: Independent and joint effects of moderate alcohol consumption and smoking on the risks of non-alcoholic fatty liver disease in elderly Chinese men
Source: PLoS One. 2017 Jul 20;12(7):e0181497. doi: 10.1371/journal.pone.0181497 (PMC5519158; doi:10.1371/journal.pone.0181497)
Supplement: S2 Table — (DOCX) [file pone.0181497.s002.docx]

Table S2 Baseline characteristics of study participants according to cigarette smoking (n=9432)

|  | Never smoking | Current smoking | Former smoking | *P* value |
| --- | --- | --- | --- | --- |
| Total number | 3825 | 3204 | 2403 |  |
| Age(years) | 66.61(7.07) | 63.67(6.35) | 66.54(6.40) | <0.0001 |
| Body Mass Index(kg/m^2^) | 24.77(3.14) | 24.19(3.23) | 24.88(3.22) | <0.0001 |
| Overweight, n(%) | 2302(60.18) | 1666(52.00) | 1502(62.50) | <0.0001 |
| Education(1/2/3) | 25.48/32.66/41.87 | 29.82/37.57/32.61 | 30.00/33.51/36.49 | <0.0001 |
| Waist circumference(cm) | 85.48(9.25) | 84.38(9.83) | 86.09(10.04) | <0.0001 |
| HDL (mmol/l) | 1.35(0.38) | 1.34(0.39) | 1.35(0.44) | 0.8665 |
| LDL (mmol/l) | 2.98(0.96) | 3.00(0.82) | 2.97(0.80) | 0.3842 |
| ALT (U/L) | 26.43(29.97) | 23.75(15.66) | 25.39(16.50) | <0.0001 |
| AST (U/L) | 26.43(20.55) | 24.67(13.68) | 25.72(11.18) | <0.0001 |
| AST/ALT | 1.15(0.46) | 1.19(0.59) | 1.14(0.41) | 0.0002 |
| Triglyceride (mmol/l) | 1.44(1.18) | 1.42(1.42) | 1.43(1.05) | 0.6824 |
| Cholesterol (mmol/l) | 4.99(0.95) | 4.98(0.99) | 4.95(0.95) | 0.4467 |
| Uric Acid (μmol/l) | 330.30(83.44) | 321.77(78.62) | 335.58(82.49) | <0.0001 |
| Systolic blood pressure (mm Hg) | 132.64(18.22) | 129.19(18.58) | 132.29(17.37) | <0.0001 |
| Diastolic blood pressure (mm Hg) | 78.58(11.03) | 77.64(11.15) | 78.42(10.82) | 0.0012 |
| Diabete Mellitus, n(%) | 846(22.12) | 550(17.17) | 545(22.69) | <0.0001 |
| History of coronary heart disease, n(%) | 968(25.32) | 680(21.23) | 854(35.55) | <0.0001 |
| History of hypertension, n(%) | 1652(43.20) | 1157(36.12) | 1212(50.46) | <0.0001 |
| History of stroke, n(%) | 192(5.02) | 157(4.90) | 227(9.45) | <0.0001 |
| NAFLD, n(%) | 1235(32.29) | 1065(33.24) | 869(36.16) | 0.0060 |
| moderate drinker, n(%) | 719(18.80) | 1562(48.75) | 787(32.75) | <0.0001 |
| Physical activity, n(%) | 3268(85.44) | 2626(81.96) | 2103(87.52) | <0.0001 |

One-Way variance test for continuous variables or Pearson’s qui-squared test for categorical variables.

Overweight was defined as BMI≥24.

Educational was categorized as1, 2 and 3, which mean low (0 to 6 years), medium (7 to 9 years), and high (≥10 years) respectively.
